# A Rare Constellation of Peripartum Cardiomyopathy Manifesting With Multiple Thromboembolic Phenomena in a 15‐Year‐Old

**DOI:** 10.1002/ccr3.73299

**Published:** 2026-08-06

**Authors:** Reuben E. Mkama, Bibiana Kazaura, Theresia E. Lutufyo, Baraka E. Mrisho, Hery O. Kimwela, Marco E. Nyakasalule, Ronald M. Costantini, Eliya Msemwa, Florence Malamso, George Assenga, Julius Kinyange, Josephine R. Laswai, Fabian M. Kamana, Godfery Malangwa, David S. Mazengo, Raymond Leiya, Andrea S. Hondo, Masuma Gulamhussein, Happiness J. Mathew, Paul P. Mwasapi, Phillip S. Muhochi, Hakolo L. Mgaya, Moa E. Kalunga, Brendan P. Shayo, Amani E. Senso, Leonia N. Ndakamaze, Tuzo Lyuu, Anna A. Sanga, Rosemary P. Minja, Deusdedit Ndilanha, Emmanuel J. Magandi, Nkulikiye Kanani, Anthony J. Gyunda, Immaculate R. Goima, Justus L. Ishengoma, Janet L. Mtenga, Flora N. Ndobho, Kigocha L. Okengo, Paschal N. Kulwijila, Pius C. Njingo

**Affiliations:** ^1^ Department of Critical Care Medicine Muhimbili National Hospital‐Mloganzila Dar es Salaam Tanzania; ^2^ Muhimbili University of Health and Allied Sciences Dar es Salaam Tanzania; ^3^ Department of Critical Care Medicine Muhimbili National Hospital‐Upanga Dar es Salaam Tanzania; ^4^ Department of Internal Medicine Muhimbili National Hospital‐Mloganzila Dar es Salaam Tanzania; ^5^ Department of Obstetrics and Gynecology Muhimbili National Hospital‐Mloganzila Dar es Salaam Tanzania; ^6^ Department of Otolaryngologists Muhimbili National Hospital‐Mloganzila Dar es Salaam Tanzania; ^7^ Department of Clinical & Anatomical Pathology Laboratory Muhimbili National Hospital‐Mloganzila Dar es Salaam Tanzania; ^8^ Department of Emergency Medicine Muhimbili National Hospital‐Mloganzila Dar es Salaam Tanzania; ^9^ Department of Anesthesiology Muhimbili National Hospital‐Mloganzila Dar es Salaam Tanzania; ^10^ Muhimbili National Hospital‐Mloganzila Dar es Salaam Tanzania

**Keywords:** anticoagulation, cardioembolic stroke, hypercoagulable state, peripartum cardiomyopathy, postpartum, pulmonary embolism

## Abstract

A 15‐year‐old postpartum primipara presented at 19 days with aphasia, right hemiparesis, chest pain, and dyspnea. Diagnostics confirmed concurrent cardioembolic stroke and pulmonary embolism secondary to peripartum cardiomyopathy (ejection fraction 29%). Managed successfully without thrombolysis using low‐molecular‐weight heparin, bromocriptine, heart failure therapy, and multidisciplinary care, achieving near complete neurological recovery.

**Key Clinical Message**

Peripartum cardiomyopathy (PPCM) hypercoagulability from pregnancy stasis and left ventricular dysfunction of ejection (LVEF ≤ 35%) drives rare dual stroke/pulmonary embolism (PE) in postpartum youth. Screen aggressively sans cardiac history—as in this 15‐year‐old case yielding full recovery. Multidisciplinary diagnosis/anticoagulation slashes thromboembolism recurrence.

## 1 Introduction

Peripartum cardiomyopathy (PPCM) is a rare, idiopathic form of dilated cardiomyopathy that arises in late pregnancy or the early postpartum period. It is defined by the development of heart failure in the absence of an alternative identifiable cause, accompanied by left ventricular (LV) systolic dysfunction with an ejection fraction (EF) consistently below 45% [1]. Although the precise etiology remains unknown, several risk factors have been implicated, including African descent, advanced or very young maternal age, pregnancy‐related hypertension, multiparity, multiple gestations, obesity, chronic hypertension, prolonged tocolytic use, and genetic susceptibility [1, 2, 3].

PPCM is associated with a substantially heightened prothrombotic risk. The estimated incidence is approximately 1 in 4000 live births, yet only about 6% of affected patients experience thromboembolic complications such as ischemic stroke or PE [4, 5]. Mortality rates range from 7% to 50%, often resulting from cardiogenic shock, thromboembolism, or arrhythmias [2]. The development of thromboembolism in PPCM is thought to arise from the combined effects of pregnancy‐induced hypercoagulability and LV dysfunction, which promotes relative blood stasis [6]. Consequently, current guidelines recommend anticoagulation for PPCM patients with severely reduced LVEF ≤ 35% to mitigate thromboembolic risk [2, 5, 7].

The simultaneous presentation of cardioembolic stroke and acute PE as the initial manifestations of PPCM—without preceding heart failure symptoms—is exceptionally uncommon and poses a major diagnostic and therapeutic challenge. The absence of standardized protocols for managing this “triple threat” (heart failure, stroke, and PE) necessitates a tailored, multidisciplinary approach. Here we present the case of a 15‐year‐old primiparous woman who presented 19 days postpartum with concurrent right basal ganglia cardioembolic stroke and pulmonary embolism, leading to a new diagnosis of PPCM. This case report highlights the critical need for high clinical suspicion for PPCM in postpartum thromboembolic events and discusses evidence‐based management strategies.

## 2 Case History/Examination

A 15‐year‐old lady was admitted to MNH‐Mloganzila following referral from Morogoro Regional Referral Hospital for cardiologist review and further management as she was diagnosed with heart failure, NYHA class IV with reduced ejection fraction, stroke, and pericardial effusion.

She was admitted to Kilosa district hospital with an obstetric history of Para 1 Living 1, 19 days post vaginal delivery with an outcome of term male baby, body weight 3 kg, cried immediately. There was no ANC card. According to the informant husband, there was no evidence of gestational diabetes, pregnancy‐induced hypertension, or excessive weight gain in her antenatal period. There was no history of addiction to alcohol, cocaine, or any other drug abuse. There is no family history of chronic illnesses like diabetes mellitus, hypertension, sickle cell disease, no history of open TB contact, or any cardiac disease.

Moreover, no complications were encountered immediately after delivery. However, a week following delivery she started presenting with episode of acute chest pain dull in nature, non‐radiating progressing in severity with time associated with episodes of difficulty in breathing, chest tightness, shortness of breath, easy fatigability and heat beat awareness, 4 days later she developed right sided weakness on both upper and lower limbs with inability to communicate well, before these complains she denied any history of fevers, weight loss, night sweats traumas or convulsions. These events prompted her to seek medical attention at a local hospital, where she was diagnosed with a lower urinary tract infection, given IV and oral medications unfamiliar to the patient or relatives. Due to the severity and deterioration of her condition, she was later referred to Morogoro RRH.

Furthermore, during her stay at Morogoro RRH, diagnostic investigations yielded critical findings: while initial labs were normal, chest x‐ray confirmed cardiomegaly, and a brain computed tomography scan (CT scan) showed ischemic changes. An echocardiogram (ECHO) revealed a severely depressed ejection fraction of 14.6% alongside mild pericardial effusion. To manage these conditions, her treatment regimen included IV furosemide 40 mg twice, Digoxin 0.25 mg once, and Junior Aspirin 75 mg once. Despite clinical interventions, her condition failed to stabilize, necessitating a referral to Muhimbili National Hospital (MNH)‐Mloganzila for specialized cardiologist review and advanced management.

Similarly, upon arrival and examination, the patient was conscious but appeared ill, moderately pale, and weak. She was dyspneic and tachypneic (22 bpm), requiring 2 L of oxygen via nasal prongs to maintain 99% saturation. Auscultation revealed fine basal crepitations. On Cardiovascular, a pansystolic murmur was noted, and the apex beat was displaced to the 6th intercostal space at the left midclavicular line. There was no peripheral edema, and other vital signs were stable. Neurological: While sensation remained intact, there was a profound loss of motor function (Grade 1/5 power) in both the right upper and lower limbs.

Upon admission to the obstetrics and gynecology ward, the patient was managed by a multidisciplinary team. Given the critical nature of the case and clinical outlook, followed by a decline in consciousness, an episode of hypotension, and unresolved respiratory distress, despite being on noninvasive minimal oxygen support, the patient was escalated to the ICU for intensive support.

Also, upon admission to the ICU, she underwent a thorough diagnostic workup and the initiation of a comprehensive therapeutic strategy. Initial labs revealed moderate anemia (Hb 7.7 g/dL), elevated D‐dimer (1406 ng/mL), raised cardiac markers (troponin 222.8 pg/mL, BNP 3470 pg/mL), moderate hypoalbuminemia (27 g/L), and mild electrolyte imbalance. Details are shown in Table 1.

**TABLE 1:** Laboratory parameters on admission.

Parameters	Reference range, adults	On admission
White blood count	4–10 K/ul	3.69 K/ul
Absolute neutrophil count	2–6.9 K/ul	2.7 K/ul
Hemoglobin	12–15 g/dL	7.7 g/dl
Platelets	150–410 K/ul	262.9 K/ul
C‐reactive protein	0–5 mg/L	4.7 mg/L
Blood urea nitrate	2.6–6.7 mmol/L	7.4mmo/L
Creatinine	50.4–98.1 mmol/L	65.4 mmol/L
International normalized ratio	0.99–1.19 s	1.62 s
Glycated hemoglobin (HbA1c)	4%–6%	5.6%
B‐type natriuretic peptide (BNP)	0–100 pg/mL	3470 pg/mL
Troponin‐I	13.8–17.5 pg/mL	222.8 pg/ml
Aspartate transaminase (IU/L)	0–34 U/L	69.62 U/L
Alanine transaminase (IU/L)	0–55 U/L	55.28 U/L
Prothrombin time (sec)	9.9–11.8 s	16.7 s
Partial thromboplastin time	22.1–28.1 s	28.3 s
D‐dimer	0–5 ng/mL	1406 ng/mL
Albumin(g/L)	35–59 g/L	27 g/L
Sodium	134–145 Mmo/L	131 Mmo/L
Potassium	3.5–5.5 Mmo/L	5.8 Mmo/L

**FIGURE 1:**
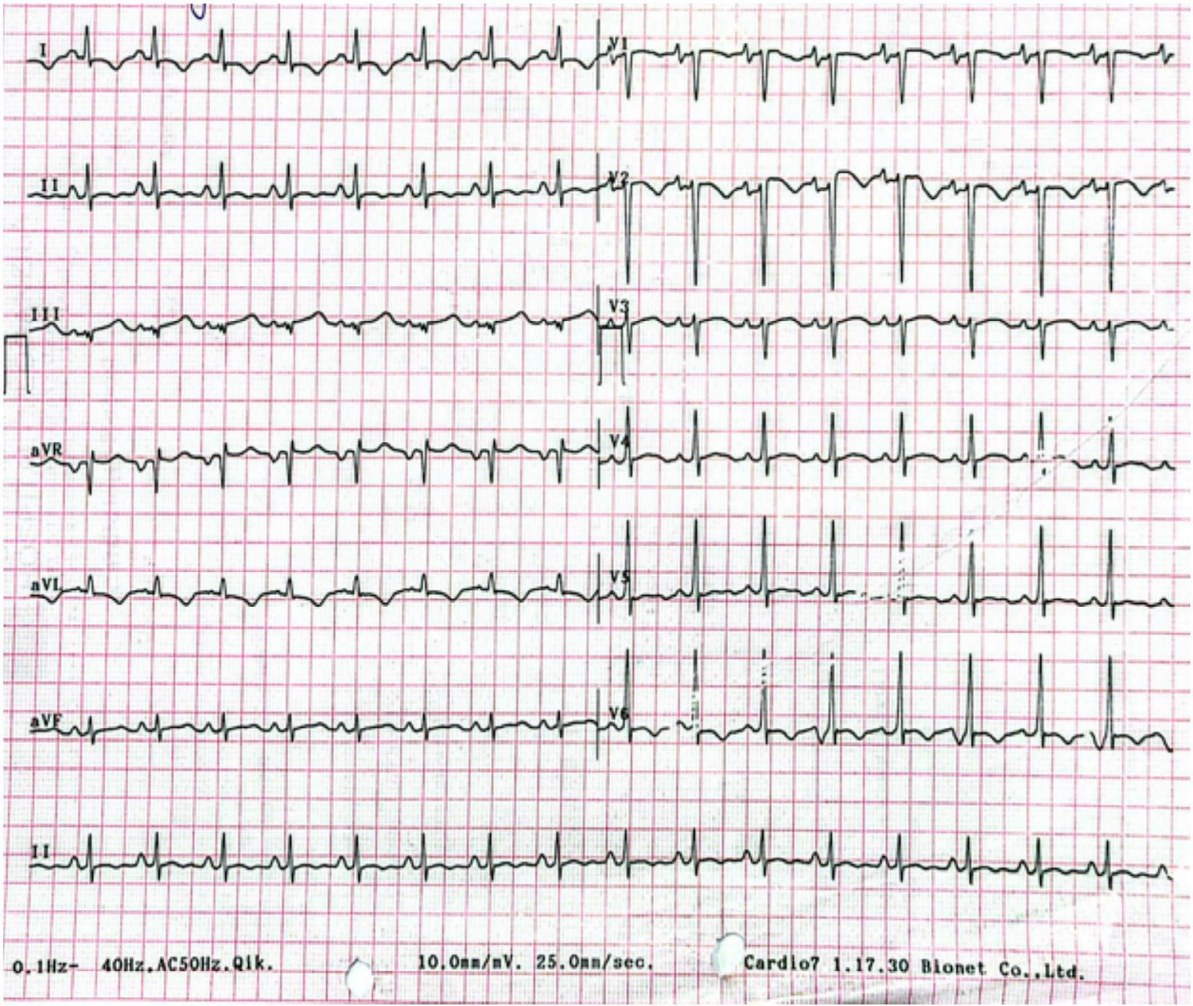
ECG–indicated sinus tachycardia and U‐waves in lead I, aVL and V6.

**FIGURE 2:**
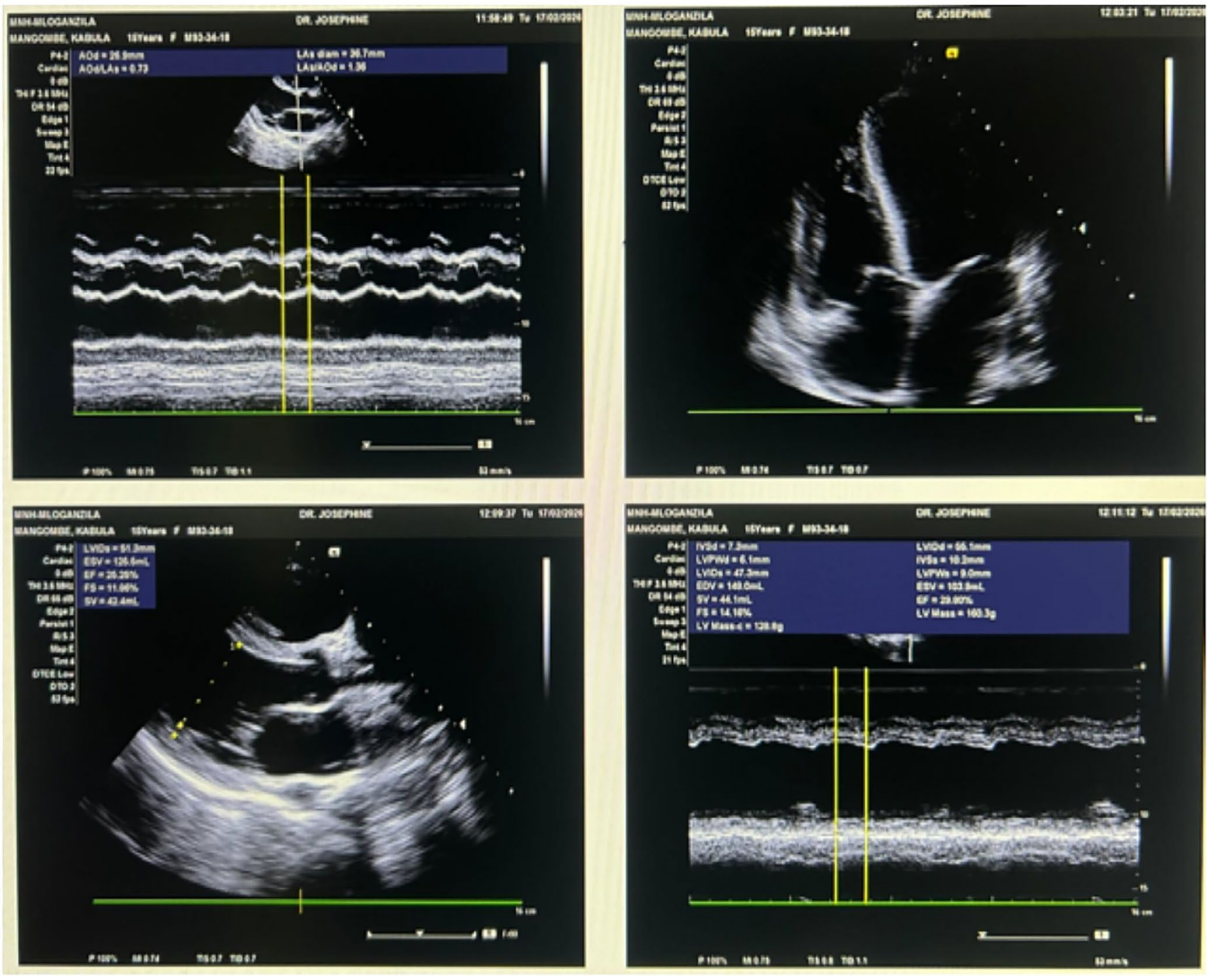
ECHO globally hypokinetic, dilated left ventricle (LV) with contrast enhancement, severe LV systolic dysfunction (LVEF 29%), and moderate mitral regurgitation.

Furthermore, the electrocardiogram (ECG) indicated sinus tachycardia and U‐waves in lead I, aVL and V6 (Figure 1). At the same time, a second comprehensive ECHO revealed a globally hypokinetic dilated left ventricle (LV) with LV ECHO contrast, exhibiting a decreased left ventricular ejection fraction (LVEF) of 29%, moderate mitral regurgitation, and a normal carotid visual Doppler assessment (Figure 2).

**FIGURE 3:**
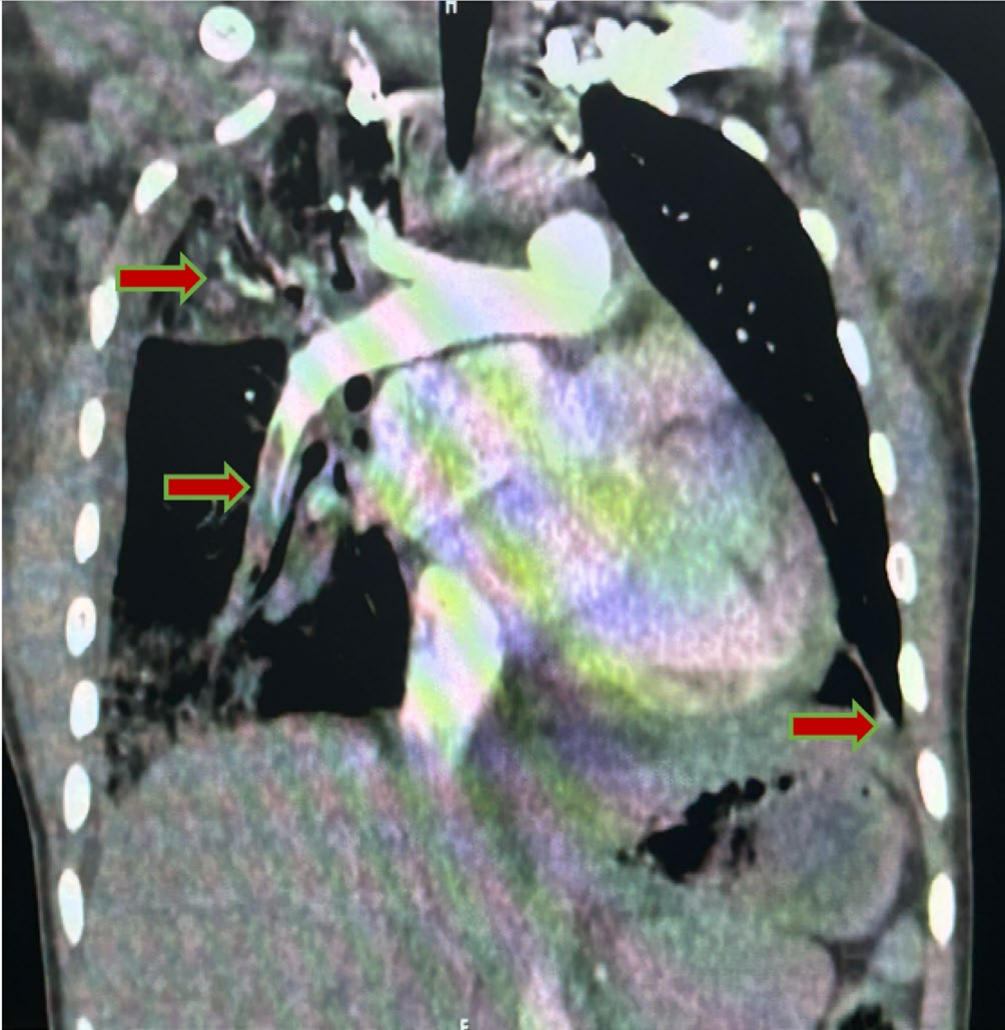
CTPA showed acute right pulmonary segmental emboli, pulmonary hypertension, bilateral pleural effusions, ground‐glass opacities, and multifocal consolidations suggestive of infection.

**FIGURE 4:**
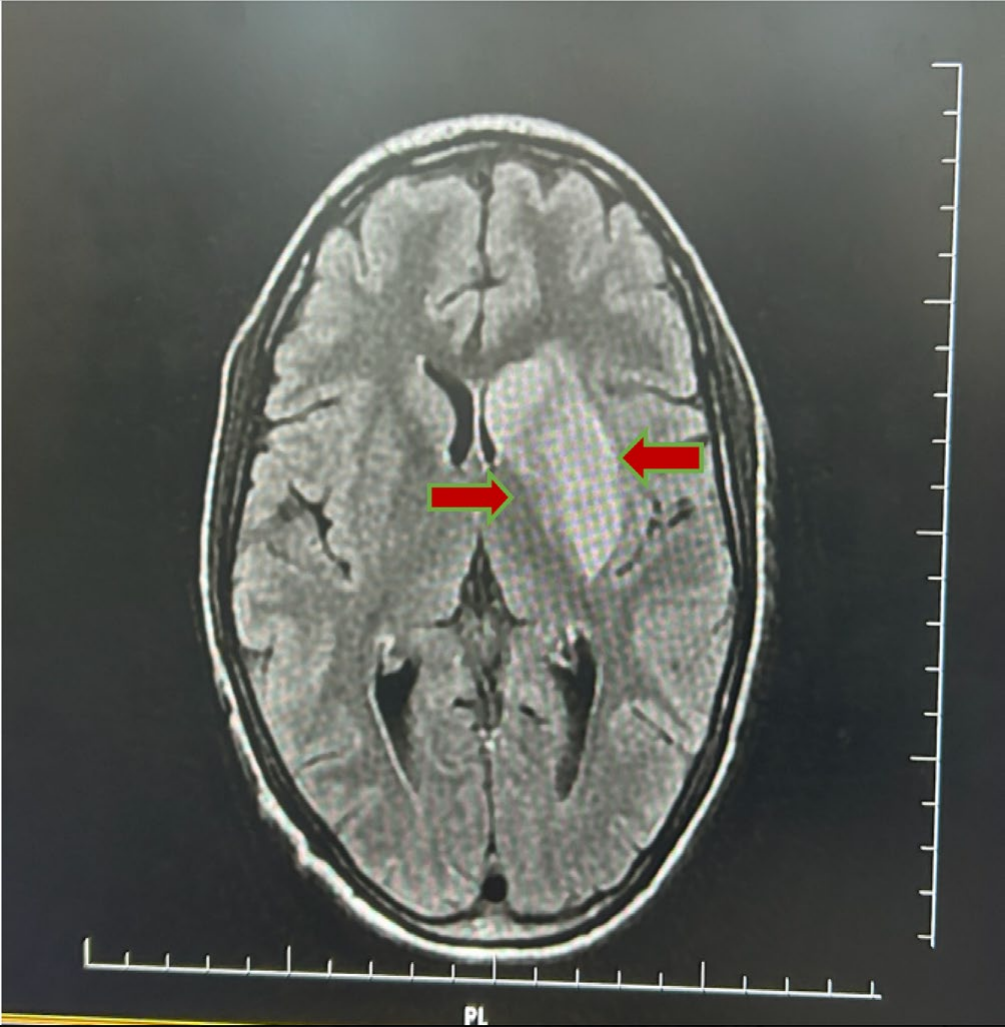
Brain MRI Scan T2 FLAIR revealed a hyperintense (bright) region in the left basal ganglia.

In terms of radiological imaging, computed tomographic pulmonary angiography (CTPA) revealed acute pulmonary emboli involving the right pulmonary segmental branch, pulmonary hypertension, bilateral pleural effusions, bilateral ground‐glass opacities, and multifocal consolidation consistent with an infectious process (Figure 3). In addition, a brain Magnetic resonance imaging (MRI) scan revealed a hyperintense (bright) region in the left basal ganglia (Figure 4).

## 3 Differential Diagnosis, Investigations, and Treatment

A clinical diagnosis was identified, centered on peripartum cardiomyopathy complicated by a cardioembolic stroke in the left basal ganglia and an acute right segmented pulmonary embolism (PE) with bilateral pleural effusion. Secondary diagnoses included pneumonia, moderate anemia, and hypoalbuminemia, alongside electrolyte disturbances consisting of mild hyponatremia and hyperkalemia.

The patient was maintained in a cardiac position and kept on oxygen therapy (10 L/min) via a non‐rebreather mask. Since she exhibited no clinical evidence of volume overload, a low dose of diuretic therapy was intentionally added to supplement her heart regimen. In addition, her management included a robust medical regimen: IV Piperacillin‐tazobactam 4.5 g thrice daily; IV Hydrocortisone 100 mg as a start dose for infection/inflammation; SC Clexane 60 mg twice daily for anticoagulation; and Sildenafil 20 mg once daily for pulmonary pressure. Cardiac and neuroprotection included Enalapril 2.5 mg once daily, Spironolactone 25 mg once daily, Clopidogrel 75 mg once daily, and Atorvastatin 40 mg nocte, IV levetiracetam 250 mg twice daily, while Bromocriptine 2.5 mg once daily was used to manage the peripartum cardiomyopathy. Supportive care comprised IV Pantoprazole 40 mg once daily, IV Paracetamol 1 g thrice PRN, and nutritional supplements (Osteocare, Nat B, Folic Acid), alongside active physiotherapy. To address her anemia, she received 2 units of packed red blood cell (PRBC) slow transfusion for 2 days, premedicated with IV Furosemide 40 mg start dose to prevent fluid overload.

## 4 Conclusion and Results (Outcome and Follow‐Up)

Following a three‐day course of intensive care, the patient demonstrated marked clinical improvement. She was successfully weaned from supplemental oxygen and maintained hemodynamic stability. Hemoglobin levels increased to 9.9 g/dL. Notably, motor function in her right limbs improved substantially, recovering to Grade 4/5 power. Following this stabilization, the patient was transferred from the intensive care unit to the general ward for ongoing management and convalescence.

Moreover, the patient received strong counseling to refrain from future pregnancies due to the high risk of relapse and cardiac deterioration. Long‐term follow‐up was planned to monitor LV function recovery via serial echocardiography and to adjust heart failure therapy accordingly.

## 5 Discussion

We report a rare case of concurrent cardioembolic stroke and pulmonary embolism as the inaugural presentation of peripartum cardiomyopathy in a 15‐year‐old primiparous woman at 19 days postpartum. This case is notable for the patient's extremely young age, the absence of any prior cardiac history or classic heart failure symptoms, and the simultaneous occurrence of arterial and venous thromboembolic events. Our discussion focuses on the pathophysiological basis of thromboembolism in PPCM, the therapeutic challenges encountered, and the rationale for our multidisciplinary management strategy.

Moreover, in keeping with prior studies, our patient's ischemic stroke and PE likely originated from a combination of LV hypokinesia‐induced blood stasis and the postpartum hypercoagulable state. Echocardiography revealed a hypokinetic, dilated LV with an EF of 29%, providing direct evidence of significant systolic dysfunction [5, 7]. Pregnancy and the puerperium are naturally hypercoagulable, with increased fibrinogen and factors VII, VIII, and X, decreased protein S activity, and acquired activated protein C resistance [6, 8]. In the presence of a poorly contracting LV, stasis within the chamber promotes left ventricular thrombus formation. Such thrombi can embolize systemically (causing stroke) or, if a patent foramen ovale is present or right heart involvement occurs, can paradoxically embolize to the pulmonary circulation [9]. Our patient's clinical profile—acute chest pain, severe respiratory distress, aphasia, and right‐sided hemiparesis within the first 2 weeks postpartum—strongly aligns with literature identifying the immediate post‐diagnosis period and the first few months after delivery as the high‐risk window for the co‐occurrence of PPCM, ischemic stroke, and PE [4, 10].

Likewise, the absence of standardized clinical protocols for managing PPCM concomitant with acute ischemic stroke and PE poses a substantial therapeutic challenge [6, 7]. Our management strategy prioritized hemodynamic stabilization and thrombus resolution while preventing recurrent thrombosis, drawing on expertise from cardiology, neurology, obstetrics, intensive care, and physiotherapy.

Additionally, evidence indicates that anticoagulation is the most effective intervention for both cerebrovascular events and venous thromboembolism in PPCM. The American Heart Association advises transient anticoagulation for LVEF ≤ 30%, while the ESC guidelines extend this recommendation to LVEF ≤ 35% [5]. Our patient presented with subacute stroke and PE without sustained hemodynamic instability or obstructive shock. We therefore selected low‐molecular‐weight heparin (LMWH) due to its advantageous safety profile, which facilitates administration during the postpartum period and breastfeeding [4, 7, 11]. Warfarin, generally regarded as safe during lactation, was considered for long‐term use. However, the timing of anticoagulation initiation required careful balancing against the risk of hemorrhagic transformation within the subacute infarct zone [11].

Furthermore, in light of the subacute presentation (beyond the 4.5‐h window for intravenous thrombolysis) and the considerable risk of intracerebral hemorrhage within the infarcted basal ganglia, systemic thrombolytics were withheld [11, 12, 13]. While systemic thrombolysis may be used during pregnancy, its application in the postpartum period is typically avoided owing to the substantial risk of life‐threatening uterine or intracranial bleeding. For patients with persistent hemodynamic instability or obstructive shock, catheter‐directed interventions or surgical embolectomy would be preferred, but these were not indicated in our stable patient [14]. Mechanical thrombectomy, although the preferred strategy for large‐vessel occlusion in eligible postpartum patients, was not required given the subacute presentation and the specific territory involved [11, 12, 13]. Instead, the patient received supportive care and antiplatelet therapy for secondary stroke prevention, consistent with established guidelines when the time window precludes thrombolysis [15, 16].

Correspondingly, heart failure was managed using an angiotensin converting enzyme (ACE) inhibitor, a potassium‐sparing diuretic, and the dopamine agonist bromocriptine. This approach aligns with the emerging consensus that prolactin inhibition, in conjunction with standard neurohormonal blockade, improves LV recovery in PPCM [6, 7]. The selection of agents was guided by their established safety profiles and compatibility with the postpartum period [7, 17, 18].

Our patient achieved near‐complete neurological recovery by discharge, attributable to early recognition, appropriate anticoagulation without hemorrhagic complications, and structured physiotherapy. This favorable outcome underscores the importance of maintaining a high index of suspicion for PPCM in any postpartum woman presenting with thromboembolic events, even in the absence of overt heart failure symptoms.

Lastly, long‐term prognosis in PPCM is variable. Recurrent heart failure occurs in approximately 25% of patients who attain recovery of LV function, compared to 50% of those with persistent systolic impairment [7]. Consequently, our patient received strong counseling to refrain from future pregnancies, as subsequent gestation carries a significant risk of relapse and further cardiac deterioration.

## 6 Limitations

Thrombolytic therapy was not administered, as the patient presented beyond the established therapeutic windows: within 4.5 h for ischemic stroke and within 48 h of symptom onset for acute PE. Although early administration yields the greatest benefit, the principal advantage in stroke lies in preserving neurological function, whereas in pulmonary embolism, it centers on rapidly restoring hemodynamic stability [19].

## 7 Conclusion

Peripartum cardiomyopathy (PPCM) represents a rare yet severe condition linked to considerable morbidity and mortality, with its etiology and pathogenesis remaining incompletely elucidated. A multidisciplinary strategy is crucial to enable prompt echocardiographic screening and enhance therapeutic optimization. PPCM is distinguished by a hypercoagulable state, and despite appropriate management, mortality rates persist at elevated levels in numerous regions.

The key lessons are: (1) Peripartum cardiomyopathy may be complicated by severe intracardiac thrombosis, which can precipitate catastrophic pulmonary and systemic thromboembolism. Therefore, a comprehensive management strategy encompassing early diagnosis, optimization of heart failure therapy, and anticoagulation is essential to mitigate adverse outcomes, alongside counseling to ensure medication adherence. (2) Patient counseling to preclude subsequent pregnancies constitutes the cornerstone of preventing recurrent heart failure. (3) Further research is warranted to evaluate and compare various management strategies for such clinical scenarios, including thrombolytic therapy and anticoagulant agents such as warfarin, heparin, and novel oral anticoagulants.

## Author Contributions

**Reuben E. Mkama:** conceptualization, writing – original draft, writing – review and editing, formal analysis, supervision. **Theresia E. Lutufyo:** conceptualization, writing – review and editing. **Baraka E. Mrisho:** conceptualization, data curation. **Ronald M. Costantini:** conceptualization, investigation. **Bibiana Kazaura:** writing – review and editing, conceptualization, investigation. **Marco E. Nyakasalule:** writing – review and editing, investigation. **Hery O. Kimwela:** data curation, conceptualization, writing – original draft. **Florence Malamso:** conceptualization, writing – original draft. **Eliya Msemwa:** conceptualization. **Fabian M. Kamana:** conceptualization, supervision. **Julius Kinyange:** conceptualization, writing – original draft. **Josephine R. Laswai:** formal analysis, validation. **Raymond Leiya:** conceptualization, formal analysis. **David S. Mazengo:** conceptualization, writing – review and editing. **Phillip S. Muhochi:** conceptualization, writing – review and editing. **Andrea S. Hondo:** conceptualization, formal analysis. **Paul P. Mwasapi:** writing – review and editing, writing – original draft. **Masuma Gulamhussein:** conceptualization, supervision. **Godfery Malangwa:** conceptualization, supervision. **Happiness J. Mathew:** conceptualization, writing – original draft. **Brendan P. Shayo:** validation, formal analysis, resources. **Moa E. Kalunga:** conceptualization, investigation. **Hakolo L. Mgaya:** resources, validation, investigation. **Amani E. Senso:** formal analysis, investigation. **Tuzo Lyuu:** visualization, writing – original draft. **Rosemary P. Minja:** formal analysis, methodology. **Leonia N. Ndakamaze:** investigation. **George Assenga:** conceptualization, investigation. **Justus L. Ishengoma:** writing – original draft, validation. **Immaculate R. Goima:** methodology, formal analysis. **Anna A. Sanga:** formal analysis, investigation. **Deusdedit Ndilanha:** methodology, formal analysis. **Emmanuel J. Magandi:** methodology, formal analysis. **Anthony J. Gyunda:** methodology, formal analysis. **Paschal N. Kulwijila:** validation, investigation. **Janet L. Mtenga:** supervision, formal analysis, validation. **Flora N. Ndobho:** writing – review and editing, writing – original draft. **Kigocha L. Okengo:** conceptualization, visualization. **Pius C. Njingo:** formal analysis, data curation. **Nkulikiye Kanani:** methodology, conceptualization.

## Funding

The authors have nothing to report.

## Consent

The patient has signed a written informed consent for the publication, which is available on request from the corresponding author.

## Conflicts of Interest

The authors declare no conflicts of interest.

## Data Availability

All data available are included in this article. Further inquiries can be directed to the corresponding author.
